# COPING STYLES AS MEDIATORS BETWEEN PURPOSE IN LIFE AND COGNITIVE FUNCTION: A LONGITUDINAL STUDY

**DOI:** 10.1093/geroni/igad104.3104

**Published:** 2023-12-21

**Authors:** Amanda Sesker, Páraic O’Súilleabháin, Martina Luchetti, Antonio Terracciano, Angelina Sutin

**Affiliations:** University of Minnesota Medical School - Duluth, Duluth, Minnesota, United States; University of Limerick, Limerick, Limerick, Ireland; Florida State University, College of Medicine, Tallahassee, Florida, United States; Florida State University, Tallahassee, Florida, United States; Florida State University College of Medicine, Tallahassee, Florida, United States

## Abstract

This study tested the association between purpose in life and coping styles and whether coping styles mediate the association between purpose and cognitive function. Longitudinal data from the Midlife Development in the United States (MIDUS) study were used to investigate associations between purpose assessed at MIDUS I (1996-1997) and coping assessed at MIDUS II (2004-2006) (N=2,386). Emotion-focused and problem-focused coping styles were then tested as mediators of the association between purpose and cognitive function (i.e., memory, executive function, and global cognition) assessed at MIDUS III (2013-2014). Higher purpose was associated with higher problem focused coping (β=.191, p<.001) and lower emotion-focused coping (β=-.227, p<.001). Coping style partially mediated the association between purpose and cognitive function about two decades later. Emotion-focused coping accounted for 14.3% of the total effect of purpose on cognitive function. Problem-focused coping mediated 16.7% of the total effect of the association of purpose on episodic memory, but did not mediate the associations between purpose with either executive function or total cognitive function. These findings provide new evidence for associations between purpose and coping, as well as links between coping and cognitive function in a large, longitudinal sample of adults. The results are consistent with the literature on purpose and better cognitive function in adulthood and suggest that coping is one mechanism linking purpose with better cognitive function.

